# An Opportune Suggestion

**Published:** 1914-11

**Authors:** 


### An Opportune Suggestion.

In a recent article in the *Urologic and Cutaneous Review,* Dr. A. Bachman, Surgeon U. S. N., gave a very pertinent suggestion in dealing with the "social evil." The author makes it very plain that the "social evil" is composed of two very distinct entities: prostitution, which he regards as a matter of morals and deals with individual conduct, and venereal diseases, which is a matter of health and deals with the question of well being. The eradication of venereal diseases, he says, will not eradicate prostitution and the suppression of prostitution, while it would lessen the source of venereal diseases somewhat, would not stamp them out. Education has positive influence in reducing prostitution, inasmuch as the majority of prostitutes come from the ignorant and weak-minded, but it has no effect in lessening the magnitude of the venereal peril.

Attempting to control prostitution, however, by preaching *suppression*, is of no avail. "The kind of women who prostitute, as long as they continue to be created, will continue to be the appeasers of the inextinguishable male desire." *Regulation* by registration and inspection neither does away with prostitution nor is it of any great benefit in preventing venereal diseases. Public opinion and legislative enactments might change the form of prostitution, but it will not change the character of the venereal diseases.

The doctor very sagaciously leaves the moral question (suppression of prostitution) to other reformers, and rather stresses the *moral* obligation of the State, municipality and the physicians to require the use of prophylactic measures. The State should compel the prostitute to furnish her customer with the necessary preventive treatment. And, in order to reach the larger foci (clandestine prostitutes) of infection, the physician should prescribe, for his male patients, the accepted prophylactic precautions.

